# Monostotic Scapular Caffey Disease: A Case Report With MRI Correlate

**DOI:** 10.7759/cureus.51533

**Published:** 2024-01-02

**Authors:** Madhu Oad, Jonathan Tu, Bilal Siddiqui, Dan Barlev, Salman Shah

**Affiliations:** 1 Department of Radiology, Nassau University Medical Center, East Meadow, USA; 2 Department of Radiology, City University of New York (CUNY) School of Medicine, New York, USA

**Keywords:** scapular hyperostosis, osteomyelitis, infantile cortical hyperostosis, periostitis, caffey disease

## Abstract

Caffey disease, also referred to as infantile cortical hyperostosis, is a self-limiting inflammatory disease of bone, typically diagnosed in infancy (ages less than five months). This disease is characterized by asymmetric, often polyostotic bony hyperostosis and expansion, with a predilection for the mandible (70-90%). We present a unique case of a two-month-old boy with monostotic scapular hyperostosis. The disease is primarily diagnosed on plain film and further evaluated with bone scintigraphy or skeletal survey to identify the extent of osseous involvement. Accompanying MR imaging is not usually obtained due to lack of specificity and diagnostic utility, and when pursued, can potentially confound the diagnosis. MR findings of this case are presented to re-iterate the benignity of this disease process and obviate the need for further invasive procedures.

## Introduction

Caffey disease is a self-limiting condition characterized by subperiosteal hyperplasia, bone expansion, and soft tissue swelling. It is typically asymmetric, often involving the mandible, followed by the clavicles, ribs, and diaphyses of long bones [1]. Mandibular involvement is considered characteristic and present in 70-90% of cases [2]. We present a case with isolated involvement of the scapula, which was subsequently imaged with MRI. This case highlights the possibility of monostotic involvement. Furthermore, the presence of MR imaging can be used as a teaching point to differentiate the findings of Caffey disease from other infectious or neoplastic etiology which can have similar radiologic features. MR imaging is usually superseded by the use of nuclear medicine, conservative management, and genetic testing in cases of Caffey disease and is avoided due to limitations in furthering the diagnosis of Caffey disease and the need for sedation in young patients. In some cases, MR can perplex the diagnosis if performed without the pre-existing differential of Caffey disease. This can prompt further unnecessary invasive workups such as biopsy/resection. In our case, we had a high suspicion of Caffey disease and MR was used to exclude other diagnoses, specifically septic arthritis and osteomyelitis. A targeted workup is necessary as the differential for Caffey disease is broad and includes osteomyelitis, non-accidental trauma, metabolic disorders, and neoplasm, which can have drastically varied management and clinical implications.

## Case presentation

**Figure 1:**
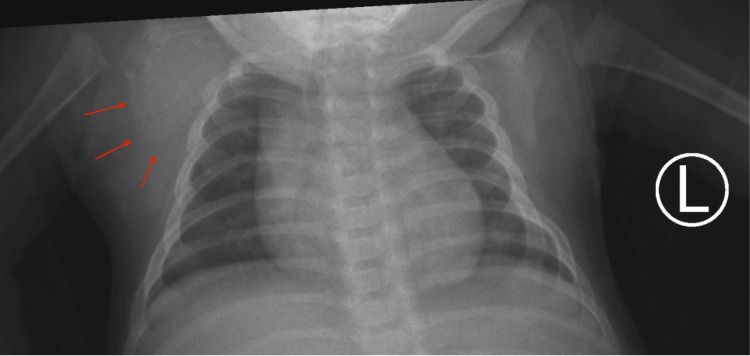
X-ray chest (frontal view) Frontal view of the chest with shallow inspiration and no acute pulmonary disease or fractures. There is hyperostosis, sclerosis, and expansion of the right scapula (red arrows).

**Figure 2:**
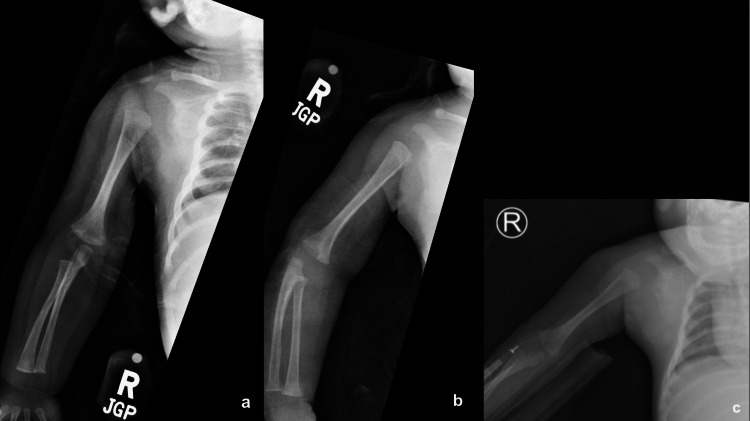
Initial frontal radiographs of the right upper extremity Initial frontal radiographs of the right upper extremity (a) and internal (b) and external (c) views of the right shoulder without evidence of acute fracture or gross dislocation.

A two-month-old male infant with uncomplicated gestation, born at 38 weeks via C-section initially presented to the emergency room with restricted right arm movement for two days. No significant family history was provided. On physical examination, the right upper extremity was generally weaker compared to the left with a full passive range of motion. No history of trauma or evidence of child abuse was noted. Laboratory values were within normal limits. Radiographs of the right upper extremity and chest were obtained without evidence of fractures or acute pulmonary disease (Figures 1, 2). Findings of right scapular hyperostosis, sclerosis, and expansion were overlooked in the initial presentation. The patient was discharged home with a neurology follow up which resulted in a prescription for physical therapy and an order for a brachial plexus MR for further neurological evaluation.

Three days following the initial ED visit, the patient returned with right arm hemiparesis, right shoulder swelling, and fever with only partial symptomatic relief with acetaminophen. While the physical exam remained unchanged, the patient had a high-grade fever of 102.5 °F and leukocytosis up to 19,000. Inflammatory markers were elevated, with an erythrocyte sedimentation rate (ESR) of 64 (reference range: <10) and a C-reactive protein level of 15.3 mg/dL (normal <10 mg/L), indicative of mild to moderate inflammation. Other workups included a negative respiratory viral panel and an ultrasound of the right shoulder which did not demonstrate a fluid collection/joint effusion. The patient was admitted with a working diagnosis of possible septic arthritis/ osteomyelitis and was started on broad-spectrum antibiotics.

**Figure 3:**
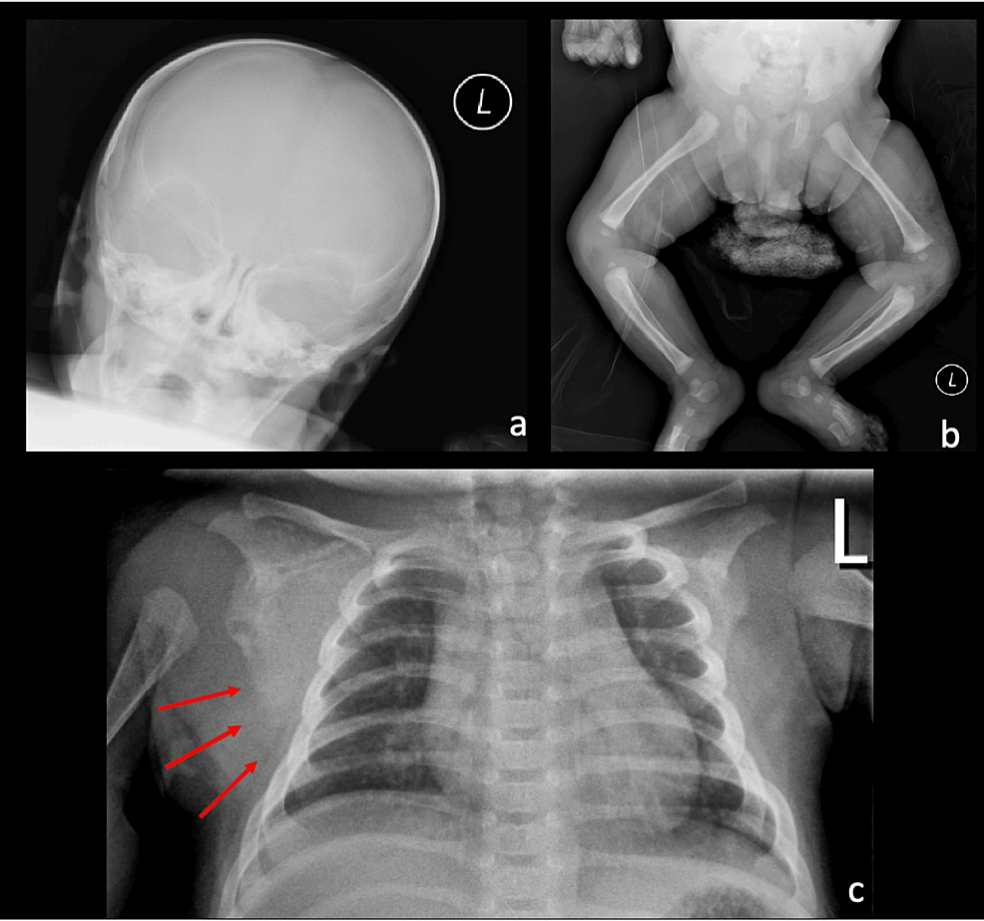
X-ray images of skull, chest, abdomen, hips and bilateral lower extremities Partial skeletal survey of the skull/mandible (a), abdomen, hips and bilateral lower extremities (b) and the chest (c). Given an available comparison of the left shoulder, there is indistinctness of the contour of the right scapula (red arrows).

**Figure 4:**
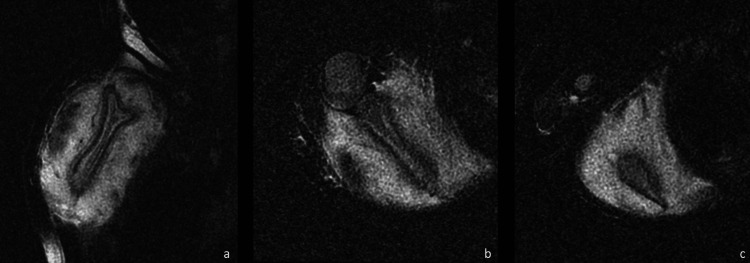
Fast spin-echo T2 weighted, fat-suppressed T2W imaging Sagittal (a) and axial (b) T2 fast spin-echo fat-sat images through the body of the superior and inferior (c) scapula are presented. There is a thickening of the cortex with subcortical edema and periostitis of the scapula without a glenohumeral joint effusion. Extensive intramuscular edema of the rotator cuff muscles (subscapularis, teres minor, supra- and infraspinatus) is noted, with relative sparing of the deltoid.

At this time, a skeletal bone survey demonstrated hyperostosis of the right scapula without additional sites of osseous involvement (Figure 3), which in retrospect, was present on initial radiographs. These findings were observed by a consulting pediatric radiologist who first suggested the diagnosis of Caffey disease. A concomitant MRI, this time requested by orthopedic surgery to rule out osteomyelitis, demonstrated prominent periostitis of the scapula with extensive surrounding intramuscular edema (Figure 4) without bony erosion or abscess formation. Given the lack of shoulder joint effusion on ultrasound and MR, as well as negative blood cultures at 48 hours, the possibility of septic arthritis was ruled out and antibiotics were discontinued. Symptomatic improvement with conservative management and NSAIDs further supported the diagnosis of Caffey disease. On follow-up, the patient continued to do well at home with complete symptomatic resolution. The parents did not wish to pursue further workup or treatment, declining nuclear medicine imaging and genetic testing.

## Discussion

This case illustrates the difficulty in the workup of musculoskeletal disease in infants, as a broad list of differentials must be considered, including congenital, traumatic, infectious, and neoplastic etiology, often without comparisons or previous history to guide the diagnosis. For example, Caffey disease with mandibular involvement can be misdiagnosed as failure to thrive due to poor oral intake [3]. In our case, an initial working diagnosis of septic arthritis was made given lab and physical exam findings, and treatment was initiated as such. There was no evidence to suggest non-accidental trauma (NAT), however, in the setting of multifocal Caffey disease, NAT would have to be considered and excluded. Clinical presentation, radiography, and response to conservative management were sufficient to make the diagnosis. Classically, this entity may present with a triad of fever, soft tissue swelling, and irritability with spontaneous resolution of symptoms requiring only supportive treatment (e.g., nonsteroidal anti-inflammatory drugs). Of note, radiographic manifestations can persist long after clinical improvement. 

Radiographic diagnosis is characterized by periosteal reaction, subperiosteal hyperostosis, bony expansion with increased cortical density and thickness, subperiosteal bone formation, and adjacent soft tissue swelling. In sporadic cases, findings are most prominent in the mandible, clavicle, and ribs but can also commonly involve the diaphyses of long bones. Additionally, the scapulae, calvarium, and ilia can be affected. The carpal and tarsal bones, phalanges and vertebral bodies are rarely involved but have been reported in the literature [2,4,5].

A radiographic skeletal survey can be used to identify the extent of osseous involvement however, bone scintigraphy with Technetium 99m-methyl diphosphonate (Tc-99m MDP) or Gallium-67 (Ga-67) is more effective in identifying areas of bone turnover not yet detectable on radiographs [3]. MRI is not the norm as it typically demonstrates non-specific periostitis and soft tissue edema, which can mimic more sinister pathology. Moreover, sedation of the infant required for MR imaging carries its own risks. Contrast-enhanced MR imaging can be performed if neoplasm or infection are higher on the differential [6] however, is typically avoided in children under two years of age due to the use of gadolinium contrast. Of note, hematopoietic bone marrow in the infant limits the use of MR imaging for the detection of osteomyelitis due to the lack of intrinsic fat signal in the medullary bone on T1 images. T2-weighted short-tau inversion recovery (T2/STIR) images can identify deep soft tissue edema which can be suggestive of bone infection. It is important to note that osteomyelitis is usually focal in nature with bony resorption, focal cartilaginous/epiphyseal involvement, and lamellated periosteal reaction. In juxtaposition, Caffey disease demonstrates circumferential spiculated cortical edema and extensive fluid signal in the adjacent soft tissues without bony destruction or resorption [7-9].

Swelling and pain associated with Caffey disease are self-limiting with subtle subsequent bony remodeling, which can be undetectable on follow-up imaging if sufficient time has passed [2]. However, on occasion, long-term complications can develop. These can range from asymmetrical or decreased bone growth with leg length discrepancy and bowing of long bones to rib fusion with subsequent scoliosis and limited chest expansion during respiration [10]. The vast majority of patients with Caffey disease have no further complications related to the disorder after early childhood, though recurrent episodes have been documented in adolescence/adulthood [3].

## Conclusions

With increased dependence on imaging for definitive diagnosis, it is imperative radiologists are cognizant of their personal and institutional limitations; we recommend obtaining specialized opinions when appropriate. Furthermore, emergency room (ER) physicians could benefit from specific training to identify bony abnormalities on plain films and obtain an early second opinion from radiology, as they can access the complete clinical history and physical exam. Together, trained ER physicians and specialized radiologists would decrease the overall rate of misdiagnosis as in the reported case. Though Caffey disease can have rare and varied presentations, MR findings associated with the disease process are infrequently discussed in a time where MR imaging is becoming a dominant modality in pediatric patients. Finally, developing a comprehensive differential based on significant clinical and radiologic findings can obviate the need for invasive and unnecessary imaging and procedures, guiding clinicians to prompt diagnosis and treatment.
